# Validation of CARE kV automated tube voltage selection for PET-CT: PET quantification and CT radiation dose reduction in phantoms

**DOI:** 10.1186/s40658-021-00373-8

**Published:** 2021-03-20

**Authors:** Natalie A. Bebbington, Troels Jørgensen, Erik Dupont, Mille A. Micheelsen

**Affiliations:** 1 Siemens Healthcare A/S, Bredskifte Alle 15, 8210 Aarhus V, Denmark; 2 Department of Clinical Medicine, Aalborg University, Søndre Skovvej 15, 9000 Aalborg, Denmark; 3 Department of Nuclear Medicine, Zealand University Hospital, Køge, Denmark; 4 Department of Biomedical Engineering, Region Zealand, Roskilde, Denmark

**Keywords:** CARE kV, kV, optimisation, PET-CT, dose, quantification

## Abstract

**Background:**

Applied tube voltage (kilovolts, kV) and tube current (milliampere seconds, mAs) affect CT radiation dose and image quality and should be optimised for the individual patient. CARE kV determines the kV and mAs providing the lowest dose to the patient, whilst maintaining user-defined reference image quality. Given that kV changes affect CT values which are used to obtain attenuation maps, the aim was to evaluate the effect of kV changes on PET quantification and CT radiation dose using phantoms.

**Method:**

Four phantoms (‘Lungman’, ‘Lungman plus fat’, ‘Esser’ and ‘NEMA image quality’ (NEMA IQ)) containing F-18 sources underwent 1 PET and 5 CT scans, with CARE kV on (automatic kV selection and mAs modulation) and in semi mode with specified tube voltages of 140, 120, 100 and 80 kV (mAs modulation only). A CARE kV image quality reference of 120 kV/50 mAs was used. Impact on PET quantification was determined by comparing measured activity concentrations for PET reconstructions from different CT scans with the reconstruction using the 120 kV reference, and dose (DLP, CTDI_vol_) differences calculated by comparing doses from all kV settings with the 120 kV reference.

**Results:**

CARE kV-determined optimal tube voltage and CARE kV ‘on’ dose (DLP) savings compared with the 120 kV reference were: Lungman, 100 kV, 2.0%; Lungman plus fat, 120 kV, 0%; Esser, 100 kV, 9.3%; NEMA IQ, 100 kV, 3.4%. Using tube voltages in CARE kV ‘semi’ mode which were not advised by CARE kV ‘on’ resulted in dose increases ≤ 65% compared with the 120 kV reference (greatest difference Lungman plus fat, 80 kV). Clinically insignificant differences in PET activity quantification of up to 0.7% (Lungman, 100 kV, mean measured activity concentration) were observed when using the optimal tube voltage advised by CARE kV. Differences in PET quantification of up to 4.0% (Lungman, 140 kV, maximum measured activity concentration) were found over the full selection of tube voltages in semi mode, with the greatest differences seen at the most suboptimal kV for each phantom. However, most differences were within 1%.

**Conclusions:**

CARE kV on can provide CT radiation dose savings without concern over changes in PET quantification.

## Background

Computed tomography (CT) scanning is known to be the greatest contributor to radiation burden associated with medical imaging examinations [1]. In positron emission tomography (PET)-CT imaging, CT is performed in addition to the PET scan, for one or more of the following purposes: ‘attenuation correction’ (AC) of PET data, ‘localisation’ of abnormal tracer uptake shown in the PET images, ‘characterisation’ of the underlying disease associated with abnormal tracer uptake or with ‘diagnostic’ intent [2, 3]. The level of CT image quality needed and therefore the radiation dose delivered to the patient can be very low for AC, localisation and characterisation scans but increases markedly when performing fully diagnostic scans [3]. National dose surveys have shown that localisation and/or characterisation (L/C) are the most common clinical reasons (termed ‘clinical purposes’) for whole-body PET-CT scans for oncology and inflammation or infection indications [2, 3]. Despite L/C level CT scans allowing relatively low CT exposure settings compared with fully diagnostic scans [2, 3], the fact that PET-CT scans cover the whole-body in many clinical applications means the CT component still contributes a significant proportion of the overall radiation dose in whole-body PET-CT [4, 5]. Given the high CT radiation burden in medical imaging, there has thus been much focus on CT dose reduction techniques.

CARE kV (Siemens Healthcare GmbH, Erlangen, Germany) is one such application, allowing dose reduction through modulation of tube current and automatic selection of the optimal tube voltage according to the size and density of the individual patient [6]. Whilst other CT dose optimisation technologies such as tube current modulation alone and iterative reconstruction have demonstrated great value in reducing CT dose, dose reduction with these methods is usually implemented through a corresponding reduction in tube current. However, with the already low tube current settings used for the common purposes of AC, localisation and characterisation in PET-CT examinations, dose reduction can be limited by the lowest tube current that can be delivered by the system, particularly in small patients or paediatrics [7]. Thus, tube voltage optimisation could be a means of achieving a lower possible radiation output from the system.

CT image quality (measured by contrast-to-noise ratio, CNR) and radiation dose are influenced by exposure settings such as tube voltage, tube current and rotation time. Tube voltage is measured in kilovolts (kV) and influences the average energy and thus penetrating power of the resulting x-rays. Radiation dose increases exponentially with kV which therefore greatly influences dose [8]. Tube current is measured in milliamperes (mA) and influences the rate at which x-rays are produced by the tube. Tube rotation time is measured in seconds and represents the time for the x-ray tube and detector unit to complete one full rotation. The product of tube current and rotation time, referred to as 'mAs' (milliampere seconds), influences the number of x-rays produced per tube rotation, and there is a linear increase in dose with mAs [9]. CARE kV requires the user to set a reference level of image quality in the scan protocol which is achieved by selecting a reference kV and mAs to be used for a standard sized (70–80 kg) patient, together with a setting for the scan type for which the dose saving is to be optimised: non-contrast-enhanced soft tissue, contrast-enhanced soft tissue or vascular scans. CARE kV uses the topogram (scout scan) for each patient, to determine their attenuation in each slice along the z-axis. This in turn allows the scanner to optimise the corresponding kV and mAs settings to provide the lowest radiation dose to each patient for the defined scan range, whilst maintaining the requested reference image quality [6]. When CARE kV is ‘on’, it automatically selects the optimal kV for that patient and scan range, which is defined by the kV and mAs combination using the lowest dose to provide the reference image quality. In addition, it optimises the dose savings for the specific scan type in terms of contrast status mentioned above. In CARE kV ‘semi’ mode, the scanner will not automatically select the optimal kV for the specific patient and scan range. Rather, the user must define a specific kV with which to scan, and the mAs is then modulated accordingly, to provide the CNR defined by the CARE kV reference kV, mAs and scan type. When CARE kV is ‘off’, the user must define the specific kV with which to scan, yet selection of CARE Dose 4D will allow the mAs to be modulated according to the CARE Dose 4D quality reference mAs setting. However, only the quality reference mAs is considered in the reference CNR for that specific kV, and modulation according to a reference kV and scan type for dose optimisation are not considered.

Several publications have already demonstrated that CARE kV can provide considerable dose savings in diagnostic CT examinations of the chest/angiography [10–13], abdomen [14, 15] and liver [16]. This suggests that large dose savings may also be made for widely performed whole-body Fluorine-18 (F-18) Fluorodeoxyglucose (FDG) PET-CT studies. However, since CT scans are often performed without contrast media in PET-CT for AC, localisation or characterisation [3], they would likely result in different relative dose savings than those already published in the literature. Furthermore, since the CT data is used for AC of PET images [17], PET-CT users may have reservations about allowing tube voltage changes in PET-CT scan protocols, given that kV changes affect the CT values used to obtain the attenuation maps, which could potentially affect PET quantification. However, Siemens PET-CT systems use a kVp-dependent formula for converting CT images to PET attenuation maps which should minimise the effect on PET quantification [17]. The aim of this study was to assess the value of CARE kV and the feasibility of its use with PET-CT, through use of phantoms to measure radiation dose savings with CARE kV and its effect on PET quantification.

## Methods

### Phantoms

**Fig. 1:**
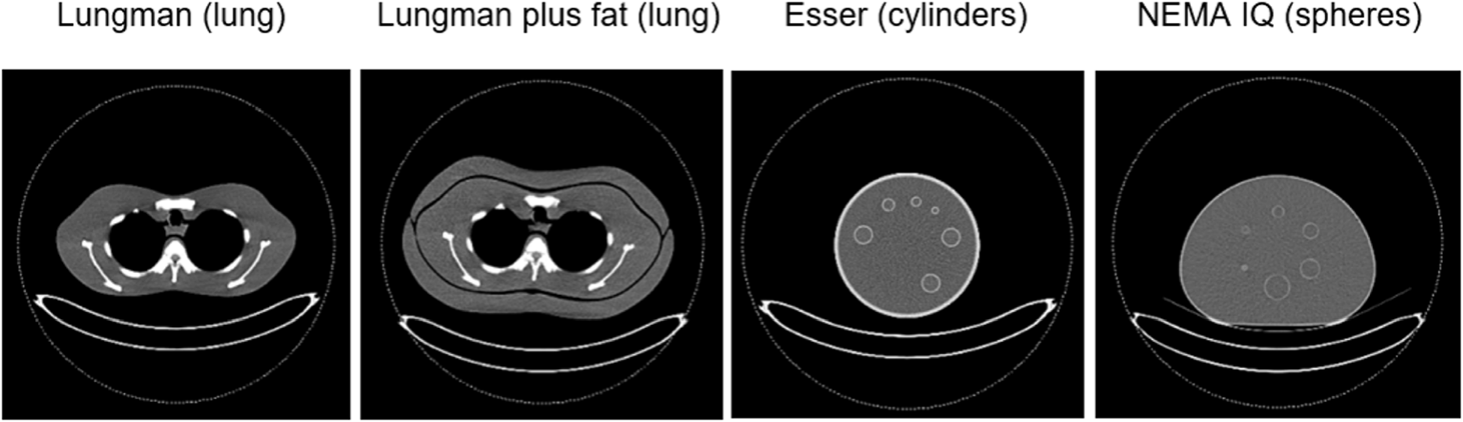
Transaxial CT images of the phantoms used in this study

A range of patient sizes and tissue densities were represented in this study through use of four phantoms: LUNGMAN N1 multipurpose chest phantom (Kyoto Kagaku, Kyoto, Japan) (*Lungman)*, LUNGMAN N1 multipurpose chest phantom plus additional fat layer (*Lungman plus fat*), Flangeless Esser PET phantom (Data Spectrum Corp., Durham, NC, USA) (*Esser*) and NEMA IEC PET body phantom (Data Spectrum Corp., Durham, NC, USA), commonly referred to as the NEMA image quality phantom (*NEMA IQ*). Transaxial CT images of the phantoms used in this study are pictured in Fig. 1.

Lungman represents a normal-sized adult male chest and is 430 mm wide, 400 mm deep and 480 mm high, with a chest girth of 94 cm. The phantom is comprised of soft tissue made of polyurethane and bone made of synthetic epoxy resin. Lungman plus fat is made by attaching anterior and posterior polyurethane plates to Lungman, each of 30-mm thickness, thereby representing an overweight adult male chest.

The Esser phantom is a water-filled acrylic cylindrical phantom of 204 mm diameter and 186 mm height used primarily for SPECT and PET image quality assessment. The phantom has a circumference of 64 cm, correlating to the waist measurement of a child from age 10 to early teens [18]. The phantom comprises six cold spheres and a compartment of rods which were included in the phantom scans but not used as part of the tracer activity evaluation. Six cylinders of height 38 mm are suspended from the phantom lid. Three cylinders have a diameter of 25 mm (volume 18.7 mL) which were used for evaluation of tracer activity concentration in this study. The remaining three cylinders have diameters and volumes of 16 mm (7.6 mL), 12 mm (4.31 mL) and 0.8 mm (1.92 mL), which were filled with activity but not used as part of the tracer activity concentration evaluation, involving accurate placement of a 1-cm^3^ volume of interest (VOI).

The NEMA IQ phantom is a water-filled acrylic phantom representing the size and shape of an adult abdomen and is used primarily for PET image quality assessment. The phantom is 300 mm wide (left to right), 230 mm deep (anterior to posterior) and 220 mm high and with a circumference of 87 cm correlates to the waist measurement of an overweight adult female [19]. It contains six spheres suspended in the central part of the phantom of diameter 10, 13, 17, 22, 28 and 37 mm. A cylindrical lung insert is also available but was not used in this study, since this would have reduced the attenuation of the phantom, and only the measured activity concentrations in the spheres were to be evaluated.

The phantoms contained F-18 FDG sources, with the activity for Lungman and Lungman plus fat contained within a cotton wool ball soaked in 2 mL of tracer solution and positioned at the base of the left lung. The activity for the Esser phantom was distributed between the 6 cylinders and the activity for the NEMA IQ phantom distributed between the 6 spheres, giving total volumes of 70 mL and 48 mL, respectively. The total activities and activity concentrations were 0.1 MBq (50 kBq/mL), 0.09 MBq (43 kBq/mL), 5.5 MBq (78 kBq/mL) and 2.72 MBq (57 kBq/mL) at the PET scan start time, for the Lungman, Lungman plus fat, Esser and NEMA IQ phantoms. None of the phantoms contained background activity since the purpose of the study was to investigate the effects of different tube voltages on quantification, rather than to study image quality.

### Measurements

All phantoms were scanned on a Biograph mCT PET-CT system with True V and 64 slice Definition A/S CT (Siemens Healthcare GmbH, Germany). For each of the four phantoms, 1 PET scan was acquired together with 5 CT scans, using CARE kV reference settings of 120 kV, 50 mAs (effective), and image quality optimised for non-contrast CT (point 3 on the sliding scale), with the phantom positioned in the centre of the gantry in *x* and *y* planes. Of the 5 CT scans performed for each phantom, 1 CT scan was performed with CARE kV *on*, whereby the scanner chose the kV and mAs combination giving the lowest dose to the patient, thus considered to be the optimal tube voltage. For the 4 remaining scans, CARE kV was operated in *semi* mode with tube voltages specified at 140, 120, 100 and 80 kV, with mAs modulated to provide the reference image quality, optimised for non-contrast-enhanced CT. A total of 5 attenuation-corrected PET reconstructions were made for each phantom, using the attenuation maps created from each of the 5 CT scans. A standard whole-body PET-CT oncology scan protocol template was used for all acquisitions, with the specific CT and PET acquisition and reconstruction protocol settings provided in Table 1, with axial scan ranges being identical for all 5 CT scans for a given phantom.

**Table 1:** (a) CT and (b) PET acquisition and reconstruction protocol settings for the phantom scans

(a)					
**CT acquisition settings**					
Scan number	1	2	3	4	5
Acquisition mode	Helical				
Organ characteristic	Abdomen				
CARE dose 4D (mAs) modulation strength	Average				
CARE kV mode	On	Semi	Semi	Semi	Semi
CARE kV reference tube voltage (kV)	120				
CARE kV reference mAs (mAs)	50				
Image quality optimisation setting	Non-contrast-enhanced CT (point 3 on sliding scale)				
Automatic tube voltage selection	On	Off	Off	Off	Off
Tube voltage used(kV)	Optimal kV determined by scanner	80	100	120	140
Tube current modulation	On				
Tube rotation time (s)	0.5				
Collimation (detector rows × mm)	16 × 1.2				
Pitch	0.8				
Axial scan length (cm)	34.7 (Lungman, lungman plus fat); 22.1 (Esser, NEMA IQ)				
**CT reconstruction settings**					
Reconstruction method	Filtered backprojection				
Kernel	B19f				
Slice thickness (mm)	5				
Increment (mm)	5				
(b)					
**PET acquisition settings**					
Acquisition mode	Step-and-shoot				
Time per bed (mins)	3				
Number of bed positions	2 (Lungman, Lungman plus fat); 1 (Esser, NEMA IQ)				
Location of PET bed position	Shoulder joints down to base of liver in Lungman and Lungman plus fat (no scanning beyond axial extent of phantom); Axial extent of Esser phantom plus 3.5 cm scanning beyond phantom lid containing cylinders; axial extent of NEMA IQ phantom (scanning exactly the axial extent)				
**PET reconstruction settings**					
Matrix	400				
Algorithm	Ultra-HD PET (time-of-flight plus point-spread-function modelling)				
Attenuation correction	Yes				
Scatter correction	Yes				
Iterations	2				
Subsets	21				
Filter (type, mm)	Gaussian, 2.5				
Slice thickness (mm)	5				

Effective mAs is used in protocols on Siemens CT systems. This is calculated by multiplying the tube current (mA) by the rotation time (s) and dividing this value by the pitch factor. With this protocol using a tube rotation time of 0.5 s and a pitch of 0.8 as shown in Table 1 together with the scanner’s minimum tube current of 20 mA, 13 is the lowest effective mAs deliverable by the system with use of this protocol.

### Analysis

When automatic tube voltage selection is not employed, it is widely accepted practice in PET-CT to use a fixed tube voltage of 120 kV, with the addition of tube current modulation. The reduction or increase in radiation dose was therefore evaluated using computed tomography dose index by volume (CTDI_vol_) and dose length product (DLP), expressed as a percentage of these dose values for the 120 kV scan performed with CARE kV in semi mode.

Effects of kV changes on PET quantification were evaluated in *syngo.via* (Siemens Healthcare GmbH, Erlangen, Germany). VOIs were assigned to each F-18 FDG source in each phantom, with a threshold at 40% of the maximum voxel value within a user-defined constraint for Lungman, Lungman plus fat and NEMA IQ phantoms, as used in clinical practice. For the Esser phantom, a 1-cm^3^ VOI was positioned centrally in the transaxial plane of the three 18.7 mm cylinders and in the distal portion of the cylinder (towards the centre of the phantom). This VOI position was chosen to reduce the effects of noise from the low sensitivity at the edge of the PET field-of-view. Percentage changes in PET quantification were evaluated by comparing measured activity concentrations for each PET reconstruction for the 140, 100 and 80 kV scans to the 120 kV scan with CARE kV in semi mode, for mean and maximum activity concentrations (kBq/mL) for each VOI.

Since CARE kV on provides an almost identical exposure and therefore dose and PET quantification results to one of the four exposures when the system is operated in semi mode (80, 100, 120 or 140 kV), additional data points are not displayed with the results for the CARE kV on setting. In order to avoid duplication of results for one of the four kV settings for each phantom, it is instead denoted in the results for semi mode which kV the scanner chose with CARE kV on, thus deemed to be the optimal tube voltage setting. It can therefore be considered that the system operation for the optimal kV setting in semi mode was identical to that with the CARE kV on setting.

## Results

### Effect on CT dose

**Fig. 2:**
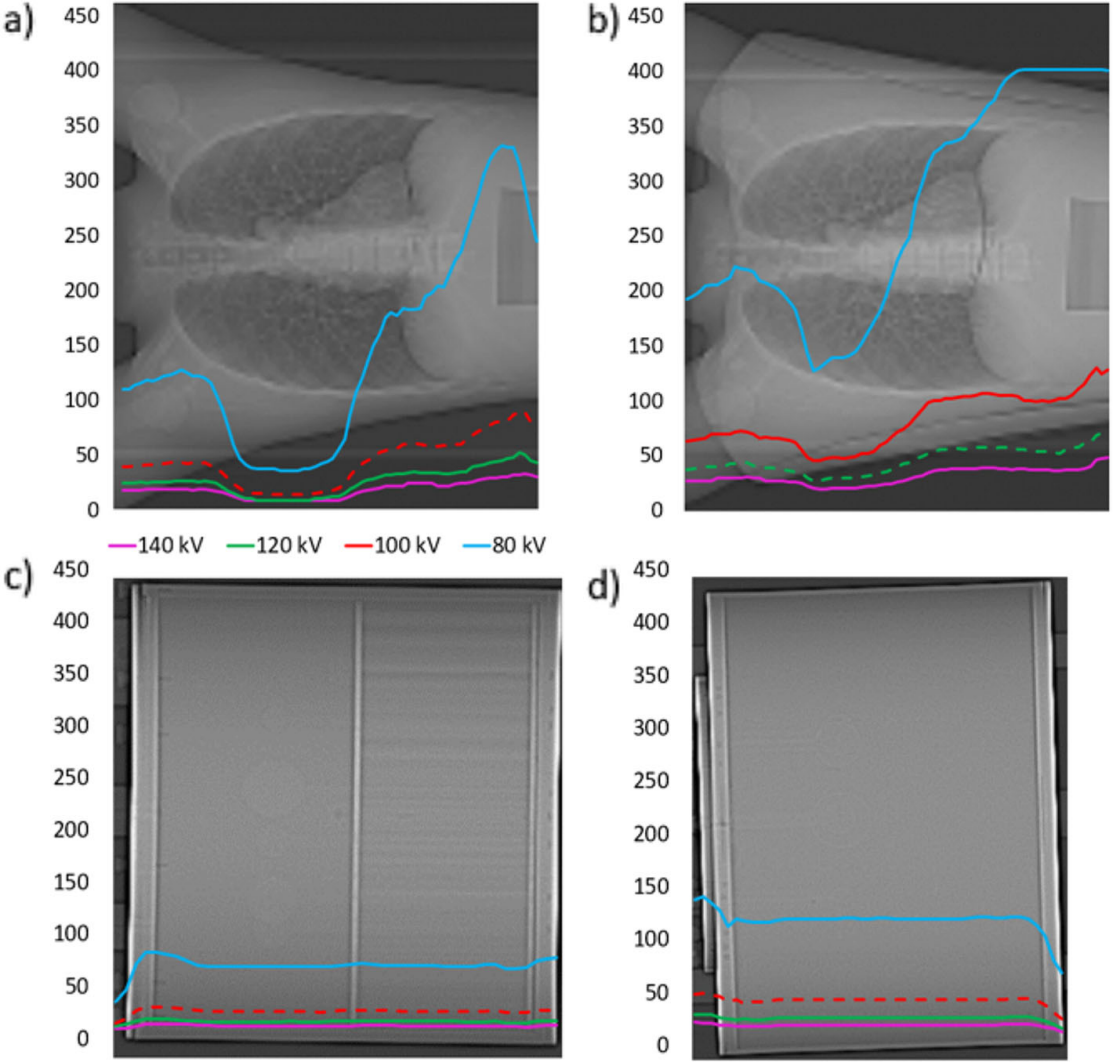
Axial mAs profiles for scans at each kV for **a** Lungman, **b** Lungman plus fat, **c** Esser and **d** NEMA IQ phantoms. Optimal kV determined by ‘CARE kV on’ mode shown with dashed profile. Minimum deliverable 13 mAs effective using selected protocol

Figures 2a to d show mAs profiles for each slice in the *z*-direction, for each phantom at each tube voltage setting.

These profiles demonstrate that as kV is reduced, mAs increases to compensate for increased image noise as expected. The plots display modest mAs increases with decreasing tube voltage from 140 down to 100 kV (approximately 2- to 2.5-fold differences between 140 and 100 kV), yet when the tube voltage is as low as 80 kV, mAs increases by around 4–6 times compared with 120 kV, in order to maintain the reference image quality. Furthermore, plot 2 a demonstrates that at 140 kV and 120 kV in Lungman, reductions in mAs at the lung were limited by the lowest deliverable tube current on this CT system.

Table 2 summarises mAs and dose (CTDI_vol_ and DLP) differences for each phantom and each kV in semi mode, relative to the 120 kV reference. Dose savings ≤ 9.3% were demonstrated with CARE kV on, whilst using a suboptimal kV in semi mode not advised with CARE kV on caused dose increases ≤ 65% compared with the 120 kV reference.

**Table 2:** Reference mAs, delivered mAs, CTDI_vol_ and DLP for each phantom and tube voltage

Phantom	Lungman				Lungman plus fat				Esser				NEMA IQ			
CARE kV status	Semi (fixed kV)				Semi (fixed kV)				Semi (fixed kV)				Semi (fixed kV)			
Tube voltage (kV)	80	100*	120 (ref)	140	80	100	120 (ref)*	140	80	100*	120 (ref)	140	80	100*	120 (ref)	140
Reference mAs (eff)	247	84	50	35	247	84	50	35	247	84	50	35	247	84	50	35
Delivered mean mAs (eff)	145	45	27	20	275	84	46	32	68	26	17	13	115	41	25	19
CTDIvol (mGy)^a^	2.99 (+ 48.8)	1.97 (− 2.0)	2.01	2.2 (+ 9.5)	5.65 (+ 64.7)	3.59 (+ 4.7)	3.43	3.57 (+ 4.1)	1.41 (+ 10.2)	1.16 (− 9.4)	1.28	1.51 (+ 18.0)	2.38 (+ 27.3)	1.81 (− 3.2)	1.87	2.13 (+ 13.9)
DLP (mGy.cm)^a^	110.2 (+ 48.9)	72.5 (− 2.0)	74	79 (+ 6.8)	206.9 (+ 65.0)	131.2 (+ 104.6)	125.4	128.3 (+ 2.3)	32.2 (+ 10.7)	26.4 (− 9.3)	29.1	35.2 (+ 21.0)	55.5 (+ 25.9)	42.6 (− 3.4)	44.1	50.2 (+ 13.8)

*The optimal tube voltage as determined by CARE kV on mode

^a^Values in brackets show percentage difference to reference (ref) dataset (120 kV scan in CARE kV semi mode)

### Effect on PET quantification

**Fig. 3:**
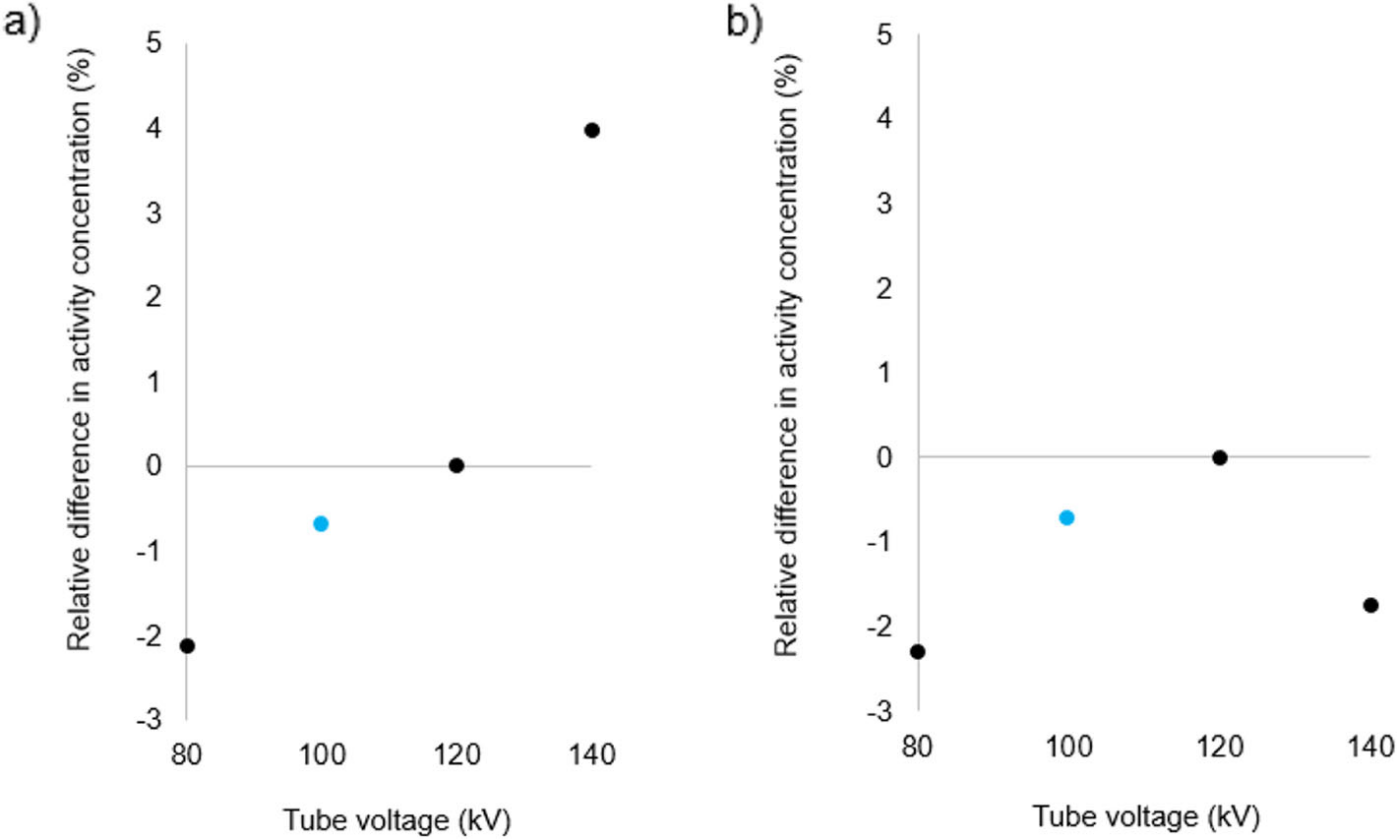
Differences in **a** maximum and **b** mean activity concentration for each kV relative to 120 kV reference in Lungman. Data points also corresponding to ‘CARE kV on’ shown with blue markers

**Fig. 4:**
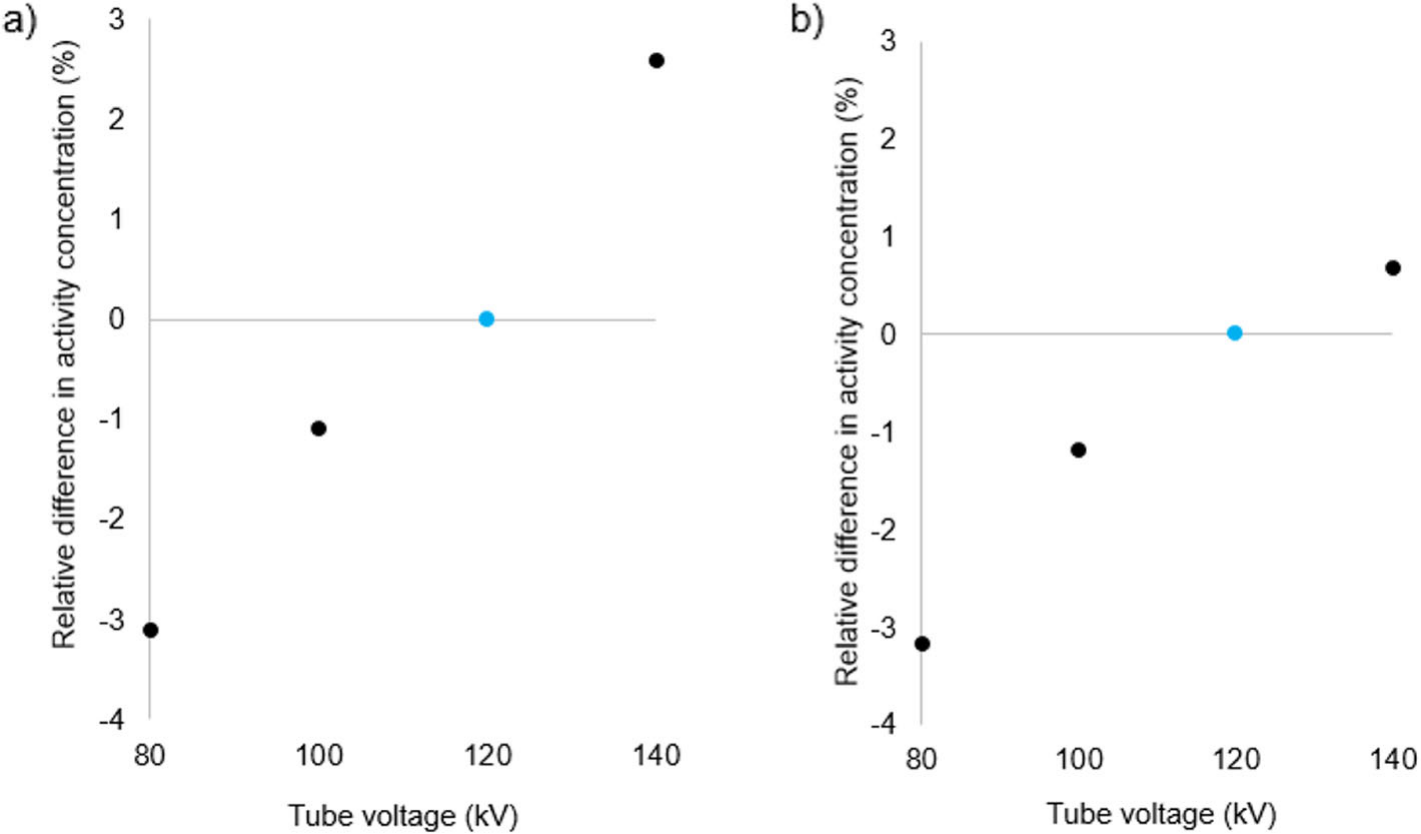
Differences in **a** maximum and **b** mean activity concentration for each kV relative to 120 kV reference in Lungman plus fat. Data points also corresponding to ‘CARE kV on’ shown with blue markers

**Fig. 5:**
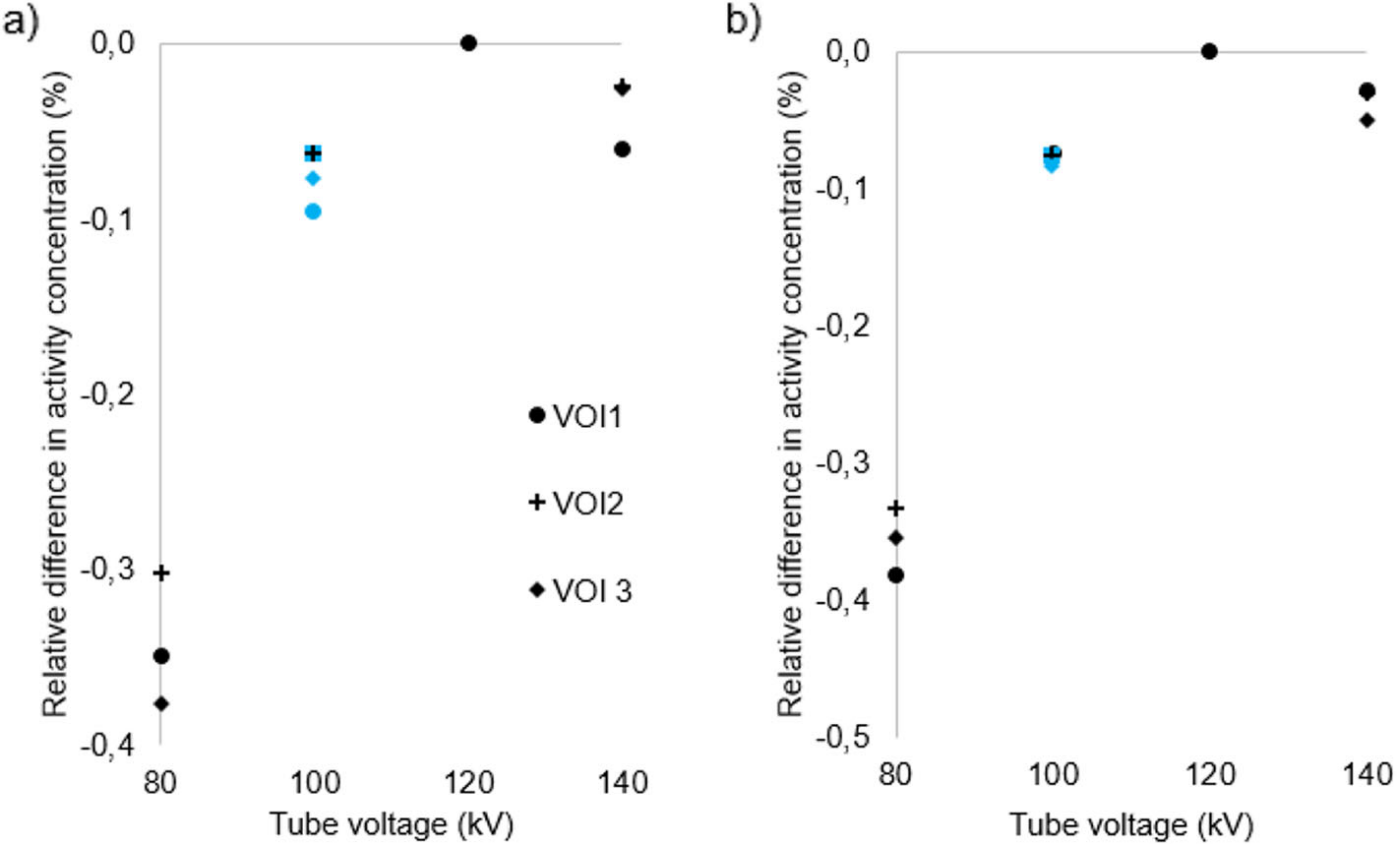
Differences in **a** maximum and **b** mean activity concentration for each kV relative to 120 kV reference in Esser phantom. Data points also corresponding to ‘CARE kV on’ shown with blue markers

**Fig. 6:**
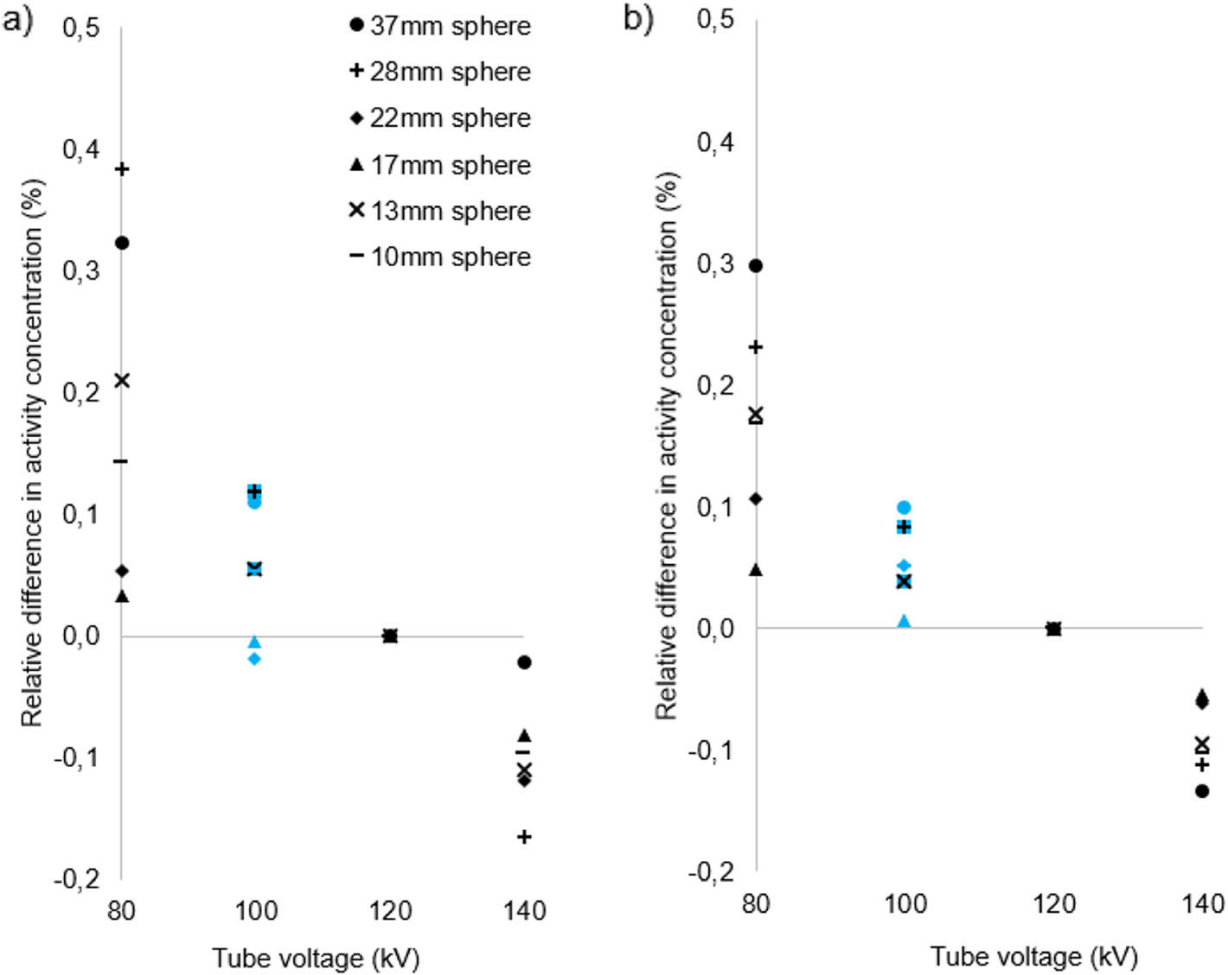
Differences in **a** maximum and **b** mean activity concentration for each kV relative to 120 kV reference in NEMA IQ phantom. Data points also corresponding to ‘CARE kV on’ shown with blue markers. There are 6 data points per kV setting corresponding to VOIs over the 6 spheres with some data points overlapping, particularly at 0.00% difference in measured activity concentration

Figures 3, 4, 5 and 6 show for each phantom, the differences in measured mean and maximum activity concentrations of the F-18 FDG sources for the scans performed at different kV with the scanner operating in CARE kV semi mode, relative to the measured activity concentrations for the 120 kV reference scan. The data points for the optimal kV as determined by CARE kV on mode are shown in blue. When scanning with the optimal tube voltage as advised by CARE kV on, only clinically insignificant differences of up to 0.7% were found in measured tracer activity concentration. The greatest difference was exhibited by Lungman using a tube voltage of 100 kV, with mean activity concentration measurement. However, differences of up to 4.0% in measured activity concentration compared with the 120 kV reference were demonstrated when using suboptimal tube voltages in CARE kV semi mode, with the greatest difference this time exhibited when measuring maximum activity concentration for Lungman with a 140-kV scan. Nevertheless, these greater differences were seen at the most suboptimal kV settings for a given phantom, and most differences for measured mean or maximum activity concentration were within 1%.

## Discussion

### Interpretation of results

This study has demonstrated CT dose savings through combined automatic kV selection and mA modulation with CARE kV compared with using the clinically accepted standard 120 kV tube voltage setting, as shown in Table 2. Scanning with the optimal tube voltage as advised by the CARE kV on setting resulted in negligible changes in quantification in the PET images as shown in Figs. 3, 4, 5 and 6. Thus, measured activity concentration and standardised uptake value (SUV) changes should not be of concern when using CARE kV on mode for whole-body FDG PET-CT examinations, when it has been estimated that the variation in maximum voxel SUV (SUV_max_) could exceed 15–20% in clinical practice [20].

This study also showed that scanning with suboptimal tube voltages specified in CARE kV semi mode had a greater influence on PET quantification and increased dose compared with scanning with the optimal tube voltage, as determined by the scanner in CARE kV on mode. In this study, scanning with a tube voltage not advised by CARE kV resulted in dose increases ≤ 65%. This demonstrates the value of CARE kV in automatically selecting the most optimal tube voltage for the individual patient. When CARE kV is on, it automatically chooses the optimal kV for that patient and scan range, determined by the kV and resulting mAs delivering the lowest dose whilst providing the reference image quality. Therefore, the CARE kV on setting should not cause an increase in dose, compared with the standard 120-kV setting. However, it could be that for a given patient and scan range 120 kV is the optimal tube voltage. In this case, the dose would not change compared with that of a 120 kV scan in CARE kV ‘semi’ mode. However, it is still an advantage to use CARE kV, since the dose saving will still be optimised for the scan type, which in this investigation was set to non-contrast-enhanced CT.

Furthermore, Fig. 2a showed how in low density structures at higher kV, the reduction in mAs can be limited by the lowest deliverable effective mAs of 13 using the selected protocol, thereby increasing dose compared with that at lower kV settings, where the reference mAs would need to be higher to maintain requested CNR. This limitation on dose saving would be even greater in paediatric patients [7] or in protocols utilising a lower reference mAs for AC, localisation or characterisation purposes.

### Explanation of results

A dose increase of 65% was seen when scanning Lungman plus fat using a tube voltage of 80 kV. When a low kV is used for a large patient or phantom, there is so much attenuation that only a very small proportion of the x-rays can penetrate the object and reach the detector, thereby greatly increasing image noise. This in turn requires an excessive increase in tube current to compensate and provide the requested reference image quality, thus resulting in a much higher dose to the patient. Figure 2 and Table 2 demonstrate a four- to six-fold increase in mAs at 80 kV compared with at 120 kV, across the range of phantoms used in this study.

Smaller patients and lower density structures provide less attenuation, meaning that fewer x-rays are absorbed within the patient and more x-rays thus reach the detector. This in turn means that for a fixed level of image quality, fewer x-rays need be produced by the tube; hence, the required mAs and dose to the patient are lower. Smaller patients and lower density structures also allow lower energy x-rays to be used thereby reducing radiation dose, whilst still providing adequate energy for enough x-rays to penetrate the patient and reach the detector to form the image. This means a lower kV and thus radiation dose can be used, even though a reduced kV may require a small increase in mAs to maintain reference image quality. Furthermore, using lower kV improves image contrast, and for a fixed CNR, the image noise would thus be greater, meaning lower mAs and dose can be used [21].

The dose savings of up to 9.3% found in this study are lower than other dose savings published in the scientific literature. Dose savings of 77% have been reported through use of CARE kV in diagnostic CT examinations of the chest/angiography [10] and 17% for abdominal scans [15], whilst dose savings of 47% have been demonstrated for liver scans employing both CARE kV and Sinogram Affirmed Iterative Reconstruction (SAFIRE) [16]. These greater published dose savings can be largely attributed to use of contrast media which was absent in this phantom study, but is commonly used for diagnostic CT performed in combination with whole-body oncology PET-CT in the Nordics [3].

### Study design

This study used phantoms rather than patients, as repeated CT scans giving additional radiation exposures were required to compare radiation dose and PET quantification of the same structures at different kV. Scanning phantoms rather than patients was therefore preferable ethically and it furthermore removed confounding effects associated with repeated patient studies such as the effects of respiratory motion.

The phantoms used in this study were intended to represent the patient sizes and densities seen in clinical practice. However, the range of phantom sizes did not cover very small or very large patient sizes sufficient for the scanner to choose 80 or 140 kV as the optimal tube voltage. At more extreme sizes where the scanner would choose 80 or 140 kV, there may be potential for greater dose savings compared with the 120 kV reference. Although some of the phantoms contained high density structures, to represent bone tissue for example, effects on tracer uptake in high density structures have not been evaluated.

Future work could therefore focus on evaluating dose savings in patients with CARE kV applied for whole-body PET-CT studies where the full range of patient sizes and densities seen in clinical practice are present and PET quantification in high density structures.

## Conclusions

This phantom study demonstrated CT radiation dose savings through scanning with the optimal tube voltage as determined by the CARE kV on setting, in PET-CT protocols utilising non-contrast CT. It further showed that using the tube voltages advised by CARE kV on mode resulted in negligible changes in the quantification of PET images. CARE kV can therefore be applied in the clinical PET-CT setting to provide a means of CT dose optimisation, which will be particularly beneficial for whole-body PET-CT examinations in which the CT component has been identified as a significant contributor to the overall radiation dose for the PET-CT examination, even for so-called ‘low dose’ localisation and characterisation purposes.

## Data Availability

The datasets used and/or analysed during the current study are available from the corresponding author on reasonable request.

## Abbreviations

AC: Attenuation correction
CNR: Contrast-to-noise ratio
CT: Computed tomography
CTDI_vol_: Computed tomography dose index by volume
DLP: Dose length product
F-18: Fluorine-18
FDG: Fluorodeoxyglucose
L/C: Localisation/characterisation
PET: Positron emission tomography
SAFIRE: Sinogram Affirmed Iterative Reconstruction
SUV: Standardised uptake value
SUV_max_: maximum voxel standardised uptake value
VOI: Volume of interest
